# EKG-Diagnostik mithilfe künstlicher Intelligenz: aktueller Stand und zukünftige Perspektiven – Teil 1: Grundlagen

**DOI:** 10.1007/s00399-022-00854-y

**Published:** 2022-05-12

**Authors:** Wilhelm Haverkamp, Nils Strodthoff, Carsten Israel

**Affiliations:** 1 Abteilung für Kardiologie und Metabolismus, Medizinische Klinik mit Schwerpunkt Kardiologie, Campus Virchow-Klinikum, Charité – Universitätsmedizin Berlin, Augustenburger Platz 1, 13353 Berlin, Deutschland; 2 Berlin Institute of Health Center for Regenerative Therapies (BCRT), Berlin, Deutschland; 3 Department für Versorgungsforschung, Fakultät VI – Medizin und Gesundheitswissenschaften, Universität Oldenburg, Oldenburg, Deutschland; 4 Klinik für Innere Medizin – Kardiologie, Diabetologie und Nephrologie, Evangelisches Klinikum Bethel, Bielefeld, Deutschland

**Keywords:** Elektrokardiographie, Digitale Gesundheitspflege, Maschinelles Lernen, Deep Learning, Künstliche neuronale Netze, Electrocardiography, Digital health, Machine learning, Deep learning, Artificial neuronal networks

## Abstract

Auch wenn die Elektrokardiographie mittlerweile ein über 100 Jahre altes diagnostisches Verfahren ist, kann die Medizin auf sie nicht verzichten. Ganz im Gegenteil, das Interesse am Elektrokardiogramm (EKG) und seine klinische Bedeutung nehmen derzeit sogar wieder zu. Dies hat nicht nur mit einer Fülle von neuen Erkenntnissen zu der elektrokardiographischen Manifestation alter und neuer kardiovaskulärer Pathologien zu tun, sondern auch damit, dass das EKG vermehrt Gegenstand von Analysen ist, die sich künstlicher Intelligenz (KI) bedienen. Die Schwächen, die der klassischen computerassistierten EKG-Auswertung anhaften, erscheinen mithilfe von KI überwindbar. Zudem scheint KI in der Lage zu sein, Informationen aus EKGs zu extrahieren, die weit über das hinausgehen, was ein Mensch leisten kann. Nicht alle Ärzte sind mit der Anwendung dieser neuen Technologie und ihren Teilbereichen, dem Machine Learning (maschinelles Lernen) und insbesondere dem Deep Learning (tiefes Lernen; wenig gebräuchlicher Ausdruck), vertraut. Die Bewertung ihrer aktuellen und zukünftigen klinischen Relevanz fällt schwer. Diese zweiteilige Übersicht beschäftigt sich mit der KI-basierten EKG-Analyse. In Teil 1 erfolgt eine Einführung in grundlegende Aspekte des Vorgehens. Teil 2, der separat publiziert wird, widmet sich dem aktuellen Stand der Forschung und bespricht die Ergebnisse verfügbarer Studien. Zudem werden möglich Szenarien der zukünftigen Anwendung von KI bei der EKG-Analyse diskutiert.

## Abstract (en)

Even though electrocardiography is a diagnostic procedure that is now more than 100 years old, medicine cannot do without it. On the contrary, interest in the procedure and its clinical significance is even increasing again. Reports on the evaluation of electrocardiograms (ECGs) with the aid of artificial intelligence (AI) are also responsible for this. Using machine learning and in particular deep learning, both AI subfields, completely new perspectives of ECG evaluation and interpretation arise. The weaknesses inherent in classical computer-assisted ECG evaluation appear to be overcome. This two-part overview deals with AI-based ECG analysis. Part 1 introduces basic aspects of the procedure. Part 2, which is published separately, is devoted to the current state of research and discusses the available studies. In addition, possible scenarios of future application of AI in ECG analysis are discussed.

Das Elektrokardiogramm (EKG), das die zyklischen Änderungen der elektrischen Aktivität des Herzens in Abhängigkeit von der Zeit auf der Körperoberfläche widerspiegelt, ist nach wie vor eines der am häufigsten eingesetzten medizinischen Diagnoseverfahren. Es sind vermutlich weit mehr als eine Milliarde EKGs, die jährlich weltweit registriert werden. Seine klinische Bedeutung und die ihm zugewiesene Aufmerksamkeit erlebt derzeit eine regelrechte Renaissance. Dies hat nicht nur mit einer Fülle von neuen Erkenntnissen zu der elektrokardiographischen Manifestation alter und neuer kardiovaskulärer Pathologien zu tun, sondern ist vor allem dadurch bedingt, dass das EKG-Signal vermehrt Gegenstand von Analysen ist, die sich künstlicher Intelligenz (KI) bedienen. Hierzu gehören Machine Learning (ML – maschinelles Lernen) und insbesondere Deep Learning (DL – tiefes Lernen, wenig gebräuchlicher Ausdruck) mittels künstlicher neuronaler Netze (KNN). Die Schwächen, die der klassischen computerassistierten EKG-Auswertung anhaften, erscheinen mithilfe dieser Technologien überwindbar. Mehr noch, es sieht so aus, als sei KI in der Lage, Informationen aus EKGs zu extrahieren, die weit über das hinausgehen, was ein Mensch leisten kann. Es ergeben sich Befunde, von denen bislang gar nicht erwartet wurde, dass sie sich mittels EKG erheben lassen [4, 21, 22]. So ist z. B. die Vorhersage der zukünftigen Entwicklung einer linksventrikulären Funktionseinschränkung oder die Vorhersage von zukünftig auftretendem Vorhofflimmern allein mithilfe eines 12-Kanal-Standard-EKGs möglich [2, 3]. Damit ergibt sich eine Erweiterung der Funktionalität des EKGs – es dient nicht mehr nur der Diagnostik, sondern wird zu einem breit einsetzbaren Screening-Instrument.

Die vorliegende Arbeit fasst den aktuellen Stand der Entwicklung zusammen und besteht aus 2 Teilen. Teil 1 beginnt mit einem kurzen Rückblick auf die Entwicklung der computerassistierten EKG-Auswertung und bespricht grundlegende Aspekte der Anwendung von KI-Techniken zur EKG-Analyse. Teil 2 gibt eine Übersicht über den aktuellen Stand der Forschung, fasst die derzeit verfügbaren Studien zusammen und bespricht die Herausforderungen, die sich in diesem Zusammenhang für uns Ärzte ergeben. Mögliche Szenarien der zukünftigen Anwendung von KI im Bereich der EKG-Analyse werden erörtert.

## Klassische computerassistierte EKG-Auswertung

Die Geschichte der computerassistierten EKG-Auswertung beginnt mit der Verfügbarkeit des EKG-Signals in digitaler Form Ende der 1950er-Jahre. Sie ist untrennbar mit Hubert Pipberger, einem Bonner Arzt und Ingenieur verbunden, der 1955 in die USA emigrierte [16]. Bereits 1961 publizierte er erste Untersuchungen zur automatischen Unterscheidung zwischen normalen und abnormen EKG [14]. Die diagnostische Genauigkeit der Verfahren war zunächst sehr beschränkt. Kritiker verglichen die computerassistierte EKG-Auswertung damals mit „Nachschwärmerei“ [17]. Technologische Verbesserungen führen dazu, dass die computerassistierte EKG-Auswertung zunehmend zu einem ernstzunehmenden Verfahren avancierte. 1980 wurde der erste kommerziell verfügbare, in ein EKG-Gerät implementierte EKG-Algorithmus vorgestellt – der 12SL-Algorithmus der Fa. Marquette [17]. Derselbe Hersteller kam Mitte der 1980er-Jahre mit einem Datenbanksystem auf den Markt (MUSE = Marquette Universal System for Electrocardiography), das eine systematisierte Archivierung von EKGs erlaubt. Die frühe und breitflächige Implementierung solcher EKG-Technologien in den Vereinigten Staaten hat dazu geführt, dass dort heute umfangreiche Datensätze mit Millionen von EKGs zu Forschungszwecken zur Verfügung stehen (Tab. 1).

**Table 1:** 

Datensatz	Lokalisation	Zeitraum	Patienten (*n*)
Telehealth Network of Minas Gerais, Brazil (Ribeiro et al. [18])	Brazil	2010–2018	1.676.384 (2.322.513 EKGs)
Mayo Clinic, Massachusetts (Kashou et al. [9])	USA	1993–2017	720.978 (2.499.522 EKGs)
Geisinger (Raghunath et al. [15])	USA	1984–2019	253.397 (1.169.662 EKGs)
Huazhong University, Wuhan (Zhu et al. [28])	China	2012–2019	71.520 (180.112 EKGs)
University of California, San Francisco (Tison et al. [25])	USA	2010–2017	36.186 EKGs

Mittlerweile verfügen nahezu alle EKG-Geräte über eine automatische EKG-Auswertung. Leider fehlt es an Standardisierung. Auch die verwendeten Dateiformate unterscheiden sich. Dieses Dilemma, das am ehesten darauf zurückzuführen ist, dass die Gerätehersteller nicht gewillt sind, freien Zugang zu den von ihren EKG-Geräten aufgezeichneten EKG-Daten zu gewähren, hat dazu geführt, dass, basierend auf der Initiative einzelner Wissenschaftler, frei zugängliche EKG-Datensätze geschaffen wurden. Einer der ersten und bekanntesten Datensätze dieser Art ist die MIT-BIH Arrhythmia Database des Massachusetts Institute of Technology (MIT), der seit Anfang der 1980er-Jahre zur Verfügung steht. Die Datenbank enthält 30-minütige, digitalisierte EKG-Ausschnitte von Langzeit-EKGs, die von Patienten des Boston’s Beth Israel Hospitals (BIH) stammen [13]. Jedes EKG ist gelabelt, d. h. mit ärztlichen Bemerkungen und Diagnosen versehen. Im Laufe der Zeit kamen weitere frei verfügbare Datensätze hinzu. Der umfangreichste frei zugängliche EKG-Datensatz ist der Datensatz der Physikalisch-Technischen Bundesanstalt in Berlin (PTB-XL), der knapp 22.000 12-Kanal-EKGs enthält [26]. Diese Datensätze finden sich heute bei PhysioNet (www.physionet.org), einer internetbasierten Forschungsplattform, die vom US-amerikanischen National Institut of Health unterstützt wird und den freien Zugriff auf diverse wissenschaftliche Datensammlungen (u. a. digitale EKG-Daten) ermöglicht (Tab. 2; [7]). Diese EKGs, die in einem einheitlichen offenen Format zur Verfügung stehen, werden nach wie vor intensiv für die Entwicklung von Algorithmen zur EKG-Auswertung genutzt – auch für Entwicklung von auf KI-basierenden Algorithmen.

**Table 2:** 

Datenbankname (Literatur)	Patienten/EKGs (*n*/*n*)	Ableitungen (*n*)	Länge (s)
MIT-BIH Noise Stress Test (Moody et al. [12])	15/15	1	22.500
MIT-BIH Arrhythmia (Moody und Mark [11])	47/48	2	1800
European ST-T Database (Taddei et al. [24])	79/90	2	7200
ICBEB Challenge 2018 (Liu et al. [10])	6877/6877	12	30
AF Classification Challenge 2017 (Clifford et al. [6])	8528/8528	1	32,5
PTB-XL (Wagner et al. [26])	18.885/21.837	12	10

Im Verlauf der letzten Jahrzehnte hat sich die diagnostische Güte der computerassistierten EKG-Auswertung zwar weiter verbessert, sie bleibt aber bis heute suboptimal [20, 27]. In 20–30 % der Fälle resultieren Fehldiagnosen, insbesondere bei der Analyse von Rhythmusstörungen. Verlassen kann sich ein Arzt auf die automatischen EKG-Auswertung nicht, eine manuelle Inspektion des EKGs mit Überprüfung des Befundes ist immer nötig. Je mehr ärztlicherseits Erfahrung in der EKG-Analyse besteht, desto mehr bleiben die automatisch erstellten Diagnosen unberücksichtigt. Die Schwächen der konventionellen automatischen EKG-Auswertung basieren in erster Linie darauf, dass die Programmierung der eingesetzten Algorithmen regelbasiert erfolgt, d. h. es wird versucht, vorgegebene EKG-Diagnosen mithilfe von programmierten Wenn-Dann-Konstruktionen abzubilden (Beispiel: Wenn die Summe der Amplitude der S‑Zacke in Ableitung V1 und der R‑Zacke in V5 oder V6 3,5 mV überschreitet, dann liegt ein eine linksventrikuläre Hypertrophie anzeigender [positiver] Sokolow-Lyon-Index vor). Viele Diagnosen lassen sich mittels solcher Regelwerke nur schlecht abbilden. Mittels KI wird heute versucht, diese Schwächen der computerassistierten EKG-Auswertung zu überwinden.

## EKG-Analyse mithilfe künstlicher Intelligenz

KI beschreibt Informatikanwendungen, die anstreben, intelligentes Verhalten zu zeigen. Ein Computer-Algorithmus wird als intelligent angesehen, wenn er in der Lage ist, selbstständig zu lernen, d. h. ohne dafür explizit programmiert worden zu sein. Es geht darum, dass die Software in Daten Muster erkennt und darauf basierend auch auf andere (fremde) Daten anwendbare Modelle kreiert. Bei der traditionellen computerassistierten EKG-Auswertung ist das anders – vom Menschen erstellte Regeln werden für die Diagnosestellung verwendet. Bei KI-basiertem überwachtem Lernen werden dem Computer Paare von Eingangsdaten und assoziierten Labels, z. B. die Zuordnung zu einer oder mehrerer vordefinierter Diagnoseklassen, vorgelegt, und der Algorithmus wird darauf trainiert, aus den Eingangsdaten, z. B. auf von Experten annotierte Pathologien, zu schließen. Weitere Formen des Lernens (unüberwachtes und bestärkendes Lernen) sind in Tab. 3 dargestellt. KI agiert damit zwar in gewisser Hinsicht selbstständig, die Regeln, nach denen KI das tut und die den Rahmen für die Entwicklung des Algorithmus festlegen, werden aber vom Programmierer (und damit von einem Menschen) vorgegeben. Im Verlauf der Entwicklung eines solchen Algorithmus werden sie, mit dem Ziel der Optimierung der Ergebnisse, vielfach angepasst. KI-Systeme ohne ein wesentliches Zutun des Menschen gibt es nicht. Dies ist vielen Laien nicht klar.

**Table 3:** 

*Überwachtes Lernen* („supervised learning“)	Die Trainingsdatensätze liegen in der Form von Paaren aus Eingabedaten und zugehörigen Annotationen bzw. Etiketten („labels“) vor. Das System lernt anhand der eingegebenen Daten neue Eingaben – entsprechend des antrainierten Lösungsweges (einem Algorithmus) – unterschiedlichen Klassen zuzuordnen (Klassifizierung) oder kontinuierliche Zielwerte vorherzusagen (Regression)
*Unüberwachtes Lernen* („unsupervised learning“)	Dem Algorithmus werden ungelabelte Trainingsdaten präsentiert. Gängige Anwendungsfälle sind das Lernen von Datenrepräsentationen und/oder das Auffinden von Strukturen in den Eingangsdaten (z. B. Clustering)
*Verstärkendes Lernen* („reinforcement learning“)	Der Computer führt Aktionen durch (z. B. Zuordnungen von Objekten zu Klassen). Je nachdem, wie letztere ausgehen, gibt es Belohnungen oder Strafen. Der Schwerpunkt dieses Verfahrens liegt auf der Entwicklung von intelligenten Lösungen für komplexe Steuerungsprobleme

**Figure 1:**
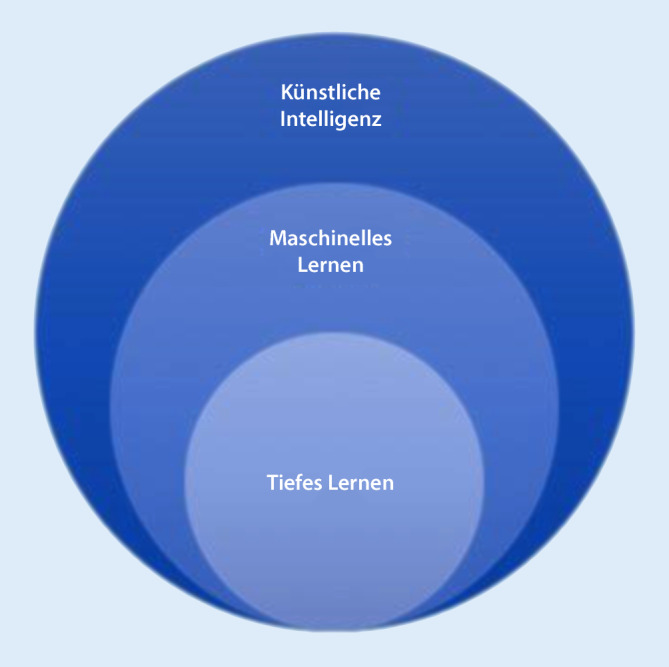

Grundsätzlich wird KI-basiertes, der Algorithmenbildung dienendes Auffinden von Mustern in Daten als ML bezeichnet. Wenn ML mithilfe vielschichtiger KNN und ausgesprochen großer Datenmengen erfolgt, wird von DL gesprochen. ML ist demnach ein Teilgebiet der KI, DL ist ein Teilgebiet von ML und KI (Abb. 1). Bei traditionellem ML werden Verfahren eingesetzt, deren Entwicklung schon in den 1950er-Jahren begann. DL ist zwar auch nicht neu, seinen Durchbruch hat das Verfahren aber erst in den letzten Jahren erlebt, im Zusammenhang mit massiven Fortschritten in der Rechnerkapazität und insbesondere dem Einsatz von hochparallelisierbaren Grafikkarten. Es wird bevorzugt bei der KI-basierten Bildanalyse, der Text- und Spracherkennung eingesetzt. Da die KI-basierte Analyse von EKGs der Bildanalyse sehr ähnelt, spielt DL auch hierbei eine wichtige Rolle [19]. Die Grundzüge des prinzipiellen Vorgehens bei der EKG-Analyse mittels ML und DL werden nachfolgend an einem fiktiven Beispiel erörtert.

Der Protagonist in unserem Beispiel ist ein junger, wissenschaftlich interessierter ärztlicher Kollege, der sich schon seit Jahren mit der Vorhersage von Vorhofflimmern beschäftigt. Das digitale Informationssystem seines Krankenhauses nutzend, hat er 2000 Patienten mit einem Alter von über 60 Jahren und Sinusrhythmus als Grundrhythmus identifiziert. Alle Patienten wurden in der Vergangenheit mehrfach stationär behandelt. Bei der Erstvorstellung war Vorhofflimmern nicht bekannt. Die Rhythmusstörung trat im Verlauf bei 15 % der Patienten (*n* = 300) auf. Nun stellt sich ihm die Frage, wie das Neuauftreten von Vorhofflimmern vorhergesagt werden kann. Mit der diagnostischen Güte des für die Vorhersage von Vorhofflimmern entwickelten CHARGE-AF-Scores (Infobox 1; [1]), der demographische und klinische Parameter (Risikofaktoren und vorhandene kardiovaskuläre Begleiterkrankungen) für die Vorhersage berücksichtigt, ist er nicht zufrieden. Der in den Publikationen mitgeteilte Kontingenzkoeffizient nach Pearson, der sog. c-Wert, der erlaubt, die diagnostische Güte in der Form der Stärke des Zusammenhangs zwischen dem Score-Wert und dem tatsächlichen Auftreten von Zielereignissen (z. B. von Vorhofflimmern) zu beurteilen, beträgt nur 0,7. Bei einem solchen Wert wird von einer nur mäßigen Fähigkeit zur Diskriminierung (bzw. einem niedrigen Vorhersagewert) gesprochen, wünschenswert wäre ein Wert von 0,8–0,9 oder > 0,9 (akzeptable bzw. sehr hohe Diskriminierungsfähigkeit). Unser junger Kollege hat sich in den Kopf gesetzt, die Vorhersage von Vorhofflimmern unter Zuhilfenahme von KI, von der er gehört hat, dass sie viel effizienter als klassische Statistik ist, zu optimieren. Es hat einen Freund, der Informatiker ist; der wird ihm helfen.

### Infobox 1 CHARGE-AF-Score

Es handelt sich um einen Score zur Vorhersage der Entwicklung von Vorhofflimmern (Alonso et al. [1]). Folgende Parameter (mit der angegebenen Effektstärke) gehen in den Score ein:

0,508 × Alter (je 5 Jahre) + 0,465 × weiße Rasse + 0,248 × Körpergröße (je 10 cm) + 0,115 × Körpergewicht (je 15 kg) + 0,197 × systolischer Blutdruck (je 20 mm Hg) − 0,101 × diastolischer Blutdruck (je 10 mm Hg) + 0,359 × gegenwärtiger Nikotinkonsum + 0,349 × antihypertensive Medikation + 0,237 × Diabetes mellitus + 0,701 × Stauungsherzinsuffizienz + 0,496 × Myokardinfarkt.

### Maschinelles Lernen

**Figure 2:**
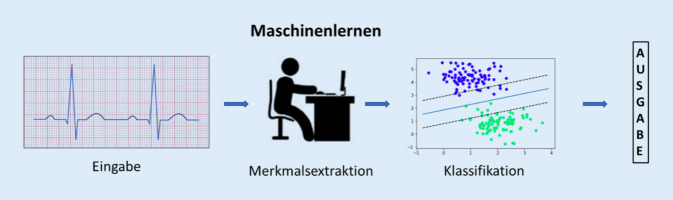

Beide beschließen, für die Entwicklung eines neuen Modells zur Vorhersage von Vorhofflimmern traditionelles ML zu verwenden. Sie planen, die Parameter des CHARGE-AF-Scores (Infobox 1) zwar zu berücksichtigen, in ihrer Verwendung aber vom Score abzuweichen. Das Alter, die Körpergröße, das Körpergewicht und der systolische und diastolische Blutdruck sollen als kontinuierliche Variablen berücksichtigt werden. Als neue kontinuierliche Variable soll die linksventrikuläre Ejektionsfraktion einfließen. Zudem glaubt unser junger Kollege, dass auch EKG-Kriterien dazu beitragen dürften, Vorhofflimmern besser vorherzusagen. Hierzu gibt es Daten in der Literatur [23]. Der Informatiker empfiehlt ihm, hierbei nicht nur gemessene Parameter wie Zeitintervalle und das Vorliegen klassischer EKG-Diagnosen (z. B. vorhandene Hypertrophiezeichen) zu benutzen, sondern das EKG-Signal intensiver auf weitere Merkmale zu untersuchen, die die Vorhersage von Vorhofflimmern eventuell verbessern könnten. Hierzu führt er diverse Transformationen des Signals durch (eine Fourier- und Wavelet-Transformation und die sog. Hauptkomponentenanalyse). Ziel dieses Vorgehens, das auch als Feature Engineering bezeichnet wird, ist es, eine besser für ML-Anwendungen verwertbare (mathematische) Repräsentation des EKG-Signals zu erhalten (Abb. 2). Letztendlich kommen sie auf 150 Variablen, die in die Analyse eingehen sollen. Bevor mit der Modellerstellung begonnen wird, unterteilt der Informatiker die 2000 EKG-Datensätze noch in einen Trainingsdatensatz und einen Testdatensatz [19]. Dies ist ein ausgesprochen wichtiger Aspekt von ML. Mithilfe der Trainingsdatensatzes (gewöhnlich 60–80 % der Datensätze) sollen Muster in den Daten entdeckt und ein Algorithmus trainiert werden, der möglichst gut Vorhofflimmern vorhersagt. Um zu prüfen, ob das Modell gut verallgemeinert (d. h., ob es auch für fremde Datensätze taugt), wird der Algorithmus später auf die Testdaten (die übrigen Datensätze) angewendet.

Die auf den Trainingsdaten basierende Optimierung des Algorithmus erfolgt eigenständig durch den Computer, der „weiß“, welche Patienten Vorhofflimmern entwickeln und welche nicht. Für die Optimierung stehen unterschiedliche ML-Technologien, wie z. B. Entscheidungsbäume, die Bayes-Klassifikation, das k‑Nearest-Neighbors-Verfahren, Support Vector Machines, aber auch (flache bzw. einfache) neuronale Netzwerke zur Verfügung (Tab. 4). Meistens werden mehrere Verfahren eingesetzt, da es nicht möglich ist, vorherzusagen, welches Verfahren den besten Algorithmus (mit der höchsten Vorhersagegenauigkeit) liefern wird. Wegweisend bei der Optimierung sind bei allen ML-Technologien fehlerhafte Vorhersagen, die in frühen Stadien der Algorithmuserstellung natürlich nicht wundern. Der Algorithmus wird anhand des berechneten Fehlers so lange vom Computer verändert (angepasst), bis die Vorhersage die an sie gestellten Erwartungen erfüllt. Diese Optimierung erfolgt häufig mithilfe eines Verfahrens, das als stochastischer Gradientenabstieg („stochastic gradient descent“) bezeichnet wird [19]. Zunächst wird für eine Menge an Beispielen ein Fehlermaß berechnet. Dann bestimmt man, in welche Richtung man die internen Parameter der Modelle verändern muss, damit sich das Fehlermaß möglichst stark verkleinert, und bewegt sich dann einen Schritt in diese Richtung. Dieses Verfahren wird für alle Trainingsbeispiele und in der Regel mehrfach durchlaufen. Die Ausgabe des endgültigen Ergebnisses erfolgt meistens in Form einer kontinuierlichen Wahrscheinlichkeit (z. B. 0 = kein Vorhofflimmern, 1 = Vorhofflimmern). Um die Ausgabe zu dichotomisieren wird gewöhnlich ein Schwellenwert festgelegt (z. B. die Festlegung, dass ab einer Wahrscheinlichkeit von 0,8 ein „Ja“, bei einer Wahrscheinlichkeit von unter 0,8 ein „Nein“ ausgegeben wird). Durch Anpassung des Schwellenwertes nach unten kann das Modell empfindlicher gemacht werden, allerdings auf Kosten einer höheren Rate an falsch-positiven Ergebnissen. Von einem guten Algorithmus wird erwartet, dass die Güte der Vorhersage (der Anteil richtiger Klassifizierungen) bei Verwendung des Testdatensatzes weitgehend mit der bei Verwendung des Trainingsdatensatzes entspricht. Wenn dies der Fall ist, wird davon gesprochen, dass der Algorithmus in der Lage ist, zu verallgemeinern (damit sollte er auch für die Klassifizierung fremder Datensätze taugen). Es kann vorkommen, dass die Vorhersage beim Trainingsdatensatz viel besser ist als beim Testdatensatz (sog. Overfitting; [19]). Anschaulich bedeutet dies, dass der Algorithmus sich während des Trainings so stark auf die Besonderheiten der Trainingsdaten eingestellt hat, dass die daraus extrahierten Muster nicht auf ungesehene Testdaten verallgemeinert werden können. Es gibt vielfältige Möglichkeiten, diesem Problem entgegenzuwirken, z. B. die Erfassung von mehr Trainingsdaten, Veränderungen in der Optimierung (Regularisierung) oder auch die Verwendung von kleineren Modellen mit weniger Modellparametern.

**Table 4:** 

*Entscheidungsbäume* („decision trees“)	Basierend auf Ausprägungen werden Modelle mit möglichen Entscheidungen konstruiert (häufiger Ansatz bei überwachtem Lernen); es entstehen Baumstrukturen mit binären Verzweigungen
*Bayessche Verfahren* („Bayesian learning“)	Der Satz von Bayes berechnet bedingte Wahrscheinlichkeiten. Die Verfahren werden eingesetzt, um Wahrscheinlichkeitsprobleme zu lösen (überwachtes Lernen). Sie sind auch bei kleiner Datengrundlage anwendbar. Beispiele: naiver Bayes-Klassifikator; Gaußscher naiver Bayes-Klassifikator; multinominaler naiver Bayes-Klassifikator; Bayessches Netz
*Instanzbasierte Verfahren*	Es handelt sich um Modelle für Entscheidungsprobleme (überwachtes Lernen), bei denen die Trainingsdaten aus Beispielen bestehen, die zunächst miteinander und später mit neuen Daten verglichen werden. Beispiel: K‑Nearest Neighbors
*Support Vector Machine*	Eine Support Vector Machine unterteilt eine Menge von Objekten so in Klassen, dass um die Klassengrenzen herum ein möglichst breiter Bereich frei von Objekten bleibt (häufiger Ansatz bei überwachtem Lernen). Das Verfahren kann als Klassifikator und Regressor verwendet werden
*Cluster-Bildungen* („clustering“)	Daten werden in Abhängigkeit von Ähnlichkeiten gruppiert und/oder nach Klassen organisiert (häufiger Ansatz bei unüberwachtem Lernen). Beispiele: K‑Means; hierarchisches Clustering

**Figure 3:**
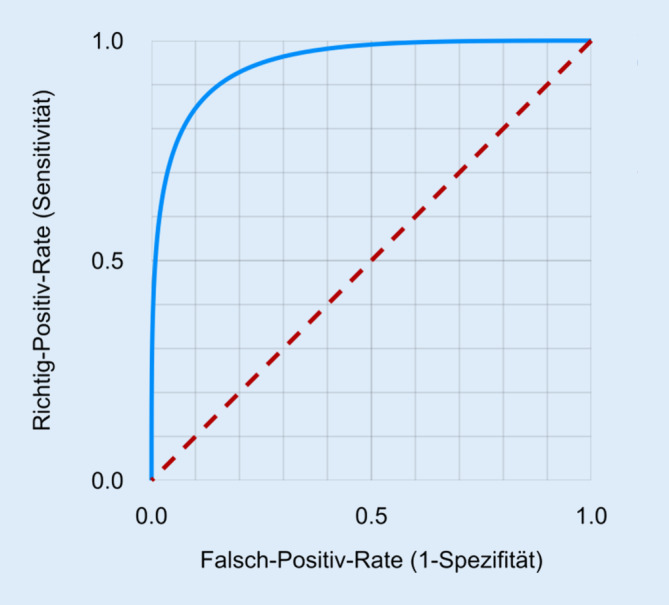

Unser junger Kollege ist ganz beeindruckt, als ihm sein Freund die Ergebnisse der Analyse zeigt. Es zeigt sich, dass sich die auf den unterschiedlichen Verfahren basierenden ML-Technologien in der Güte der Vorhersage unterscheiden. Der Informatiker belehrt ihn, dass die Bewertung letzterer bei KI-Verfahren nicht anhand des c‑Wertes, sondern anhand von „receiver operating characteristic“(ROC)-Kurven erfolgt (Tab. 5). Mitgeteilt wird meistens die Fläche unterhalb der ROC-Kurve („area under the curve“, AUC; Abb. 3). Er gibt an, wie gut das Modell zwei Klassen trennt – und entspricht in etwa dem oben diskutierten c‑Wert. Unser junger Kollege ist mit einer AUC von maximal 0,81 fürs Erste zufrieden, auch wenn es sich insgesamt mehr Diskriminierungsfähigkeit erhofft hat. Er nimmt sich vor, das Modell weiter zu verbessern. Zwischenzeitlich beginnt er mit der Planung einer Studie, die der Validierung des neuen Algorithmus dienen soll [8]. Die prospektiv angelegte Studie soll überregional bei etwa 5000 Patienten durchgeführt werden. Sie soll die Frage klären, ob der Algorithmus zur Vorhersage von Vorhofflimmern auf fremde Daten und Umgebungen übertragbar ist, denn die oben erwähnten Testdaten entstammen typischerweise der gleichen Verteilung wie die Trainingsdaten und sind daher nur bedingt aussagekräftig für die Leistungsfähigkeit des Algorithmus auf ungesehenen Daten.

**Table 5:** 

*AUC-Wert* (AUC = „area under the curve“, Fläche unterhalb der Kurve)	Der AUC-Wert gibt die Fläche unterhalb der Grenzwertoptimierungskurve, der sog. ROC-Kurve (Beispiel siehe unten) wieder. Er spiegelt die Fähigkeit eines Tests zur Diskriminierung zweier Personen bzw. Populationen. Das heißt, es wird gemessen, ob kranke und gesunde Personen anhand der Risikobewertung unterschieden werden können. Die Sensitivität und Spezifität unterliegen typischerweise einem Trade-off, d. h. eine erhöhte Sensitivität wird mit einer niedrigeren Spezifität erkauft. Die Abwägung kann mittels des Schwellenwerts getroffen werden. Das Durchlaufen aller möglichen Schwellenwerte ergibt die ROC-Kurve – der AUC-Wert ist demnach eine schwellenwertfreie Metrik und daher eine beliebte Wahl im maschinellen Lernen
*Sensitivität*	Anteil der Test-positiven Personen unter allen Erkrankten einer Stichprobe, d. h. die Wahrscheinlichkeit, mit einem diagnostischen Test die Kranken auch als krank zu identifizieren. Eine hohe Sensitivität wird angestrebt, wenn eine Erkrankung mit hoher Sicherheit ausgeschlossen werden soll
*Spezifität*	Anteil der Test-negativen Personen unter allen Nichterkrankten einer Stichprobe, d. h. die Wahrscheinlichkeit, mit einem diagnostischen Test Nichterkrankte korrekt zu identifizieren. Eine hohe Spezifität wird angestrebt, wenn eine Erkrankung mit großer Sicherheit bestätigt werden soll

Das Projekt hat viel Zeit gekostet; 3 Monate sind ins Land gegangen. Dem jungen Kollegen war zu Beginn des Projektes nicht klar, wie mühsam das Vorgehen sein würde, insbesondere bei der Aufbereitung der in die Analyse eingehenden Daten. Es hat während dieser Zeit viel über Statistik und Mathematik gelernt. In einfacherer Form hätte er das Projekt auch mit klassischer Statistik, d. h. mithilfe einer multivariaten Regressionsanalyse durchführen können. Um 150 Variablen in die Modellkonstruktion einzubeziehen, ist ML aber das geeignetere Verfahren. Darüber hinaus wollte er nicht einfach eine Patientenstichprobe untersuchen, sondern ein übertragbares Modell zur Vorhersage von Vorhofflimmern entwickeln. Bei der Durchführung des Projektes hat ihn erstaunt, das ML weit davon entfernt ist, Zauberei zu sein. Der Mensch beeinflusst sehr, was im Zusammenhang mit der Anwendung von KI herauskommt.

### Tiefes Lernen mithilfe von künstlichen neuronalen Netzen

Bei der Arbeit an seinem KI-Projekt ist unser junger Kollege auf neue Studien gestoßen, die zeigen, dass es auch allein anhand eines EKG bei Sinusrhythmus möglich ist, das zukünftige Auftreten von Vorhofflimmern vorherzusagen [3, 5]. In diesen Studien wurde KI basierend auf DL eingesetzt (eine Besprechung dieser Arbeiten erfolgt in Teil 2 dieser Übersicht). Er ist fasziniert von diesen Ergebnissen und beschließt, sich diesem Thema intensiver zu widmen.

**Figure 4:**
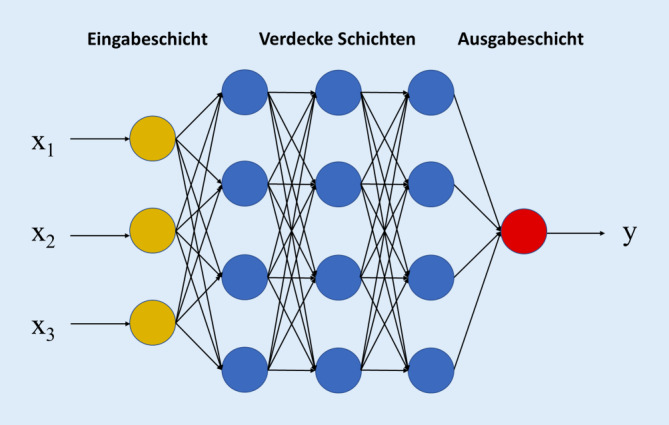

DL ist der ML-Teilbereich, der auf eine Modellbildung mithilfe von KNN beruht. Das Vorgehen erfolgt in Anlehnung an die Informationsverarbeitung und -speicherung, wie sie in zerebralen Neuronenstrukturen abläuft (Abb. 4; [19]). KNN sind aus in Schichten angeordneten Informationsverarbeitungseinheiten (sog. Knoten bzw. Neuronen) aufgebaut. Unterschieden werden können eine Eingabeschicht, verborgene Schichten und eine Ausgabeschicht. Wenn das Netz aus vielen Schichten besteht, wird auch von einem tiefen Netz gesprochen, wie es bei DL Anwendung findet. Die Übergabe von Daten an das KNN erfolgt über die Eingabeschicht, die Ausgabeschicht stellt das endgültige Ergebnis dar. Wie bei traditionellem ML erfolgt Letzteres meistens in Form von kontinuierlichen Wahrscheinlichkeiten bzw. mit Dichotomisierung. In den verborgenen Schichten werden die eingegebenen Informationen vielfältig zerlegt und schrittweise verarbeitet. Ein maßgeblicher Aspekt dieser Informationsverarbeitung ist die Gewichtung der eingehenden Daten, die schon in der Eingabeschicht beginnt. Damit bewertet das Netz die Relevanz der Information. Wenn die Summe aus dem Datenwert und Gewicht einen Schwellenwert überschreitet, erfolgte seine Weitergabe an das nächste Neuron. Mit dem Zweck der Optimierung des Modells bzw. Algorithmus durchlaufen die Daten das Netzwerk nicht einmal, sondern mehrfach, nicht selten hundertfach. Man spricht in diesem Zusammenhang auch von Iterationen. Das, was die Optimierung der Algorithmen bei DL-basiertem überwachtem Lernen vorantreibt, ist – wie bei traditionellem ML – die Maximierung des Ausmaßes der Übereinstimmung zwischen der Vorhersage durch das Modell und der tatsächlichen Zuordnung der eingegebenen Daten.

Der Hauptunterschied zwischen traditionellem ML und DL ist fließend, liegt aber in der Regel darin, dass die Modellbildung bei DL, im Gegensatz zu ML, bei dem aufwändig vorverarbeitete und damit strukturierte Daten eingegeben werden, direkt Modelle auf unstrukturierten Rohdaten als Eingangsgröße trainiert werden. Das kann natürliche Sprache, ein Bild oder ein Video oder eine Zeitreihe sein. Bei der DL-basierten EKG-Analyse besteht die Eingabe aus reellen Zahlen, die durch Abtasten des physiologischen EKG-Signals erzeugt wurden. Jeder Datenpunkt stellt die Signalamplitude für einen bestimmten Zeitpunkt dar. Beispielsweise ergeben sich bei einem 10-sekündigen 12-Kanal-EKG bei einer Abtastrate von 500 Hz insgesamt 5000 Datenpunkte mit jeweils 12 Eingangskanälen, die als Eingabe dienen. Diese Eingabe wird in den Neuronen verarbeitet. Jedes Neuron in einer Schicht mit jedem Neuron in der nächsten Schicht zu verbinden, ist zwar die allgemeinste Form der Datenverarbeitung mithilfe neuronaler Netze, sie ist aber nicht besonders effizient, da nicht geordnete Strukturen entlang der Zeitachse verwendet werden. Bei der KI-basierten Verarbeitung von EKG-Rohdaten werden bevorzugt sog. konvolutionale neuronale Netzwerke verwendet, die erstmals bei der KI-basierten Bildverarbeitung eingesetzt wurden und eine parametereffizientere Datenrepräsentation, die an diese Symmetrie des Problems (in diesem Fall Translationsequivarianz) angepasst ist, liefern [19].

Wie die in den Daten enthaltenden Informationen auf die einzelnen informationsverarbeitenden Einheiten aufgeteilt werden, ist nicht nachvollziehbar. Das Wissen ist demnach auch nicht mehr bedeutungsmäßig benennbar, es ist subsymbolisch repräsentiert. Im Gegensatz zu DL ist dies bei traditionellem ML eher möglich – die Parameter sind mehr oder weniger bekannt, es wird von symbolischer Wissensrepräsentation gesprochen. Auch wie DL die Signalmerkmale auswählt, die zu einer Ausgabe führen, ist für den Menschen nicht nachvollziehbar. KNN werden daher auch als „black boxes“ bezeichnet. Vor dem Hintergrund, dass dies die Akzeptanz von DL bei Anwendern oft beeinträchtigt, wird gegenwärtig verstärkt versucht, KI erklärbarer zu machen. Dieser Aspekt wird in Teil 2 dieser Übersicht noch einmal aufgegriffen.

Mittlerweile gibt es hunderte sich im Detail hinsichtlich der Informationsverarbeitung unterscheidende KNN-Arten. Zu den am häufigsten angewendeten KNN-Arten gehören klassische vorwärtsgerichtete KNN, rekurrente KNN und konvolutionale KNN (Tab. 6). Nicht selten werden sie kombiniert angewendet, was zu sehr komplexen Netzwerkarchitekturen führen kann. Auch eine Kombination mit ML-Verfahren kann erfolgen.

**Table 6:** 

*Vorwärts gerichtetes neuronales Netz* („feed forward neuronal network“)	Neuronales Netzwerk, das keine Rückkopplungen aufweist, d. h. die Signale laufen von der Eingangsschicht immer nur in Richtung der Ausgangsschicht. Dazwischen befindet sich mindestens eine verborgene Schicht
*Rekurrentes neuronales Netz* („recurrent neuronal network“)	Bei rückgekoppelten (rekurrenten) Netzen gibt es Neuronen, deren Ausgänge mit Neuronen derselben oder einer vorangegangenen Schicht verbunden sind. Solche Rückkopplungen ermöglichen einem neuronalen Netz ein dynamisches Verhalten und statten es gewissermaßen mit einem Gedächtnis aus
*Konvolutionales neuronales Netz* („convolutional neuronal network“)	In mehreren Schichten, die mit Filtern lupenartig analysiert werden, findet eine hierarchisch geordnete Mustererkennungen statt. Der Begriff Konvolution (Faltung) beschreibt eine mathematische Prozedur mit fundamentaler Bedeutung bei der Signalverarbeitung: Zwei Signale werden mittels Faltung zu einem dritten kombiniert. Einsatz vor allem in der Bildanalyse und der Zeitreihenanalyse (z. B. EKG-Analyse)

Für den sinnvollen Einsatz von DL ist eine ausgesprochen große Anzahl von Datensätzen notwendig. Unser junger Kollege staunt nicht schlecht, als er hört, wie groß die EKG-Datensätze sind, auf die andere Arbeitsgruppen zurückgreifen können (Tab. 1). Er sieht keine Möglichkeit, in überschaubarer Zeit an die für eine auf DL basierende EKG-Analyse notwendigen Datensätze zu kommen. In seinem Krankenhaus gibt es leider keine EKG-Datenbank. Er ist erleichtert, als er hört, dass auch die Möglichkeit besteht, ein EKG-Modell an frei verfügbaren großen Datensätzen (Tab. 2) vorzutrainieren, um es dann mithilfe zahlenmäßig kleinerer Datensätze speziellen Fragestellungen anzupassen (sog. Transfer-Lernen). Er hat beschlossen, sich für eine digitale Akquirierung von EKGs in der täglichen Krankenhausroutine und die Anschaffung einer EKG-Datenbank einzusetzen.

## Schlussfolgerungen

ML und DL sind Verfahren, die derzeit immense Fortschritte machen. Sie sind immer mehr dabei, sich auch im Bereich der Medizin zu Standardverfahren zu entwickeln. Das relativ einfach verfügbare EKG-Signal scheint sich für eine KI-basierte Analyse, insbesondere für auf DL mit KNN basierenden Analysen, besonders gut zu eignen. Ärzte, die KI-Verfahren nutzen, sollten auf jeden Fall eine Vorstellung von dem haben, was hinter KI steckt – sowohl hinsichtlich der sich ergebenen Möglichkeiten als auch bezüglich der zu beachtenden Grenzen und Limitationen. Wichtig ist es, zu realisieren, dass KI keine Zauberei, sondern ein Produkt von Menschen ist, die sich der Hilfe von Computern bedienen. In Teil 2 dieser Übersicht werden die aktuell verfügbaren Studien besprochen.
