# Erratum zu: EKG-Diagnostik mithilfe künstlicher Intelligenz: aktueller Stand und zukünftige Perspektiven – Teil 1: Grundlagen

**DOI:** 10.1007/s00399-022-00870-y

**Published:** 2022-05-31

**Authors:** Wilhelm Haverkamp, Nils Strodthoff, Carsten Israel

**Affiliations:** 1 Abteilung für Kardiologie und Metabolismus, Medizinische Klinik mit Schwerpunkt Kardiologie, Campus Virchow-Klinikum, Charité – Universitätsmedizin Berlin, Augustenburger Platz 1, 13353 Berlin, Deutschland; 2 Berlin Institute of Health Center for Regenerative Therapies (BCRT), Berlin, Deutschland; 3 Department für Versorgungsforschung, Fakultät VI – Medizin und Gesundheitswissenschaften, Universität Oldenburg, Oldenburg, Deutschland; 4 Klinik für Innere Medizin – Kardiologie, Diabetologie und Nephrologie, Evangelisches Klinikum Bethel, Bielefeld, Deutschland

**Erratum zu:**

**Herzschr Elektrophys 2022**

10.1007/s00399-022-00854-y

In der Originalversion des Beitrags stand der Hinweis zu Teil 1 im Untertitel. Dieser Hinweis wurde nun in den Haupttitel eingefügt.

Der Originalbeitrag wurde korrigiert.

